# A thin section micromorphology photomicrographs dataset of the infilling of the Sennacherib Assyrian canal system (Kurdistan Region of Iraq)

**DOI:** 10.1016/j.dib.2023.109319

**Published:** 2023-06-15

**Authors:** Stefano Costanzo, Luca Forti, Daniele Morandi Bonacossi, Andrea Zerboni

**Affiliations:** a Earth Sciences Department “A. Desio”, University of Milan, Milano, Italy; b Department of Humanities and Cultural Heritage, University of Udine, Udine, Italy

**Keywords:** Micromorphology, Geoarchaeology, Late holocene, Mesopotamia, Assyrian empire, Land use

## Abstract

Here we present a compendium of 212 photographs of archaeological soils and sediments thin sections (micrographs) from the backfill of the Sennacherib Assyrian canal system of Northern Mesopotamia. The micrographs were produced using an optical petrographic microscope (Olympus BX41) mounting a digital camera (Olympus E420) for image acquisition. The dataset is composed of two folders containing (1) every micrograph in full resolution JPEG, and (2) a PDF file with scale bars and brief captions for each one. The dataset represents a photographic comparison collection for individuals working on similar geoarchaeological contexts and can be used for the composition of figures in novel publications, as well as being the first example of published large compendium for shared use in the field of archaeology.

**Specifications Table**

**Table:** 

Subject	Archaeology; Earth-Surface Processes
Specific subject area	Geoarchaeology of arid lands, paleoenvironmental reconstruction, land use, thin section micromorphology
Type of data	Image
How the data were acquired	The soil thin sections were observed employing an optical petrographic microscope (Olympus BX41) mounting a digital camera (Olympus E420) for image acquisition.
Data format	Raw Analyzed
Description of data collection	The original undisturbed samples were collected from sections of archaeological excavations. Microscope observation and micrograph photography were carried out in the laboratory facility of the Earth Sciences Department “A. Desio” of the University of Milan.
Data source location	The data were collected in Kurdistan Region of Iraq from three locations. 1) Khinis canal site 133, 36.737647°N, 43.414882°E 2) Khinis canal site 834, 36.726125°N, 43.433732°E 3) Jerwan aqueduct site 900, 36.670245°N, 43.392145°E The thin sections are stored at the Earth Sciences Department “A. Desio” of the University of Milan.
Data accessibility	Repository name: Zenodo Data identification number: https://doi.org/10.5281/zenodo.7801619
Related research article	Forti, L., Costanzo, S., Compostella, C., Garna, G., Morandi Bonacossi, D., Zerboni, A. (2023). The geoarchaeological investigation on the defunctionalisation of an Assyrian canals system reveals late Holocene land use transitions in Northern Mesopotamia. *The Holocene*. https://doi.org/10.1177/09596836221145395

## Value of the Data

• The dataset is useful because it is a large photographic compendium of microscopic natural and anthropogenic diagnostic micro-pedofeatures from a geographic and archaeological context that has received limited geoarchaeological attention despite the prolific archaeological endeavors in the region.
• Other geoarchaeologists and archaeological scientists may benefit from the dataset, as it provides graphic reference for many identified, explained and dated [1] natural and anthropogenic features and processes connected with the use, abandonment and repurposing of the Assyrian canals of Northern Mesopotamia.
• Data can be reused as a mere study reference or as source of material for the composition of novel images in original manuscripts, textbooks, and teaching courses.

## 1 Objective

This dataset accompanies research that explores the complexity of the socioeconomic transformations of the human communities in Northern Mesopotamia. Within the project, geoarchaeological fieldwork is carried out to understand the processes involved in the natural and anthropogenic transformations of the landscape [1]. Among these, the creation of extensive canal systems is the most prominent [2].

In this context, the published research [1] tackles the paleoenvironmental significance of the infilling of some traits of the Sennacherib canal system. Therein, it is shown how every stratigraphic feature is the outcome of specific processes tied both ways with climatic/environmental changes and major shifts in land use. Among the analyses employed for the study, thin section soil micromorphology had a prominent role in disclosing pedogenetic peculiarities such as, but not limited to, hydromorphism, bioturbation, colluviation, and traces of pastoralism. Contextually, a large micrographs dataset was created. Here we present it, in order to provide graphic reference for individuals working on similar topics. We suggest that the sharing of photomicrographs datasets of archaeological soils and sediments will positively support archaeological micromorphological research, because existing atlases do not cover the great variability of observed pedofeatures.

## 2 Data Description

Data is organized in two main files, plus an additional .txt file containing instructions for navigating them.

### 2.1 File 1: Micrographs.zip

**Fig. 1:**
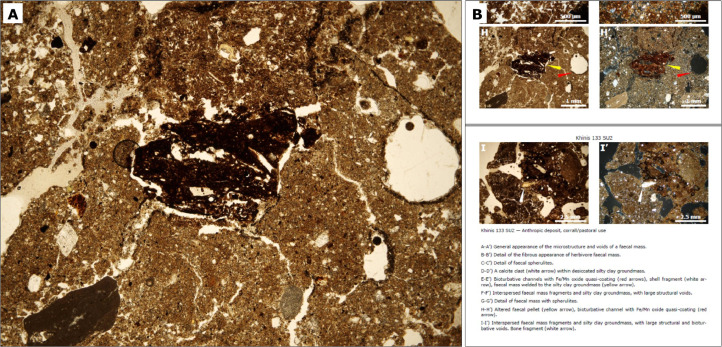
(A) Example of a full resolution unaltered .jpeg photomicrograph with no scale, as it appears in the dataset. (B) Excerpt example of the .pdf file containing the plane polarized light and cross polarized light photomicrographs with alphabetic reference to the .jpeg dataset, scale, occasional graphic additions and brief captions.

This contains the primary data. It is a .zip compressed folder containing full-resolution photographic JPEG files (micrographs) (Fig. 1A), with folder names corresponding to the name of each subset as they appear in the .pdf file (File 2) containing the interpretation of the data. Each micrograph measures 2560 × 1920 pixels with a resolution of 314 dpi and occupies approximately 1 MB of storage space. Every shot is presented with a Plane Polarized Light (PPL) and Cross-Polarized Light (XPL) version. PPL micrographs are characterized by a slightly yellow hue caused by the microscope's light source; we present them unmodified in order to avoid compression alteration.

### 2.2 File 2: Micrograph captions.pdf

This is a .pdf file containing scaled-down versions of each micrograph with name and scale bar, subdivided by Site and Stratigraphic Unit (SU) (Fig. 1B). Micrographs are presented in two columns, with each line representing the PPL and XPL version of the same shot. At the end of each group of micrographs belonging to a certain Site and SU, brief descriptions are provided to report highlights and notable features.

### 2.3 File 3: Read me.txt

This is a .txt file containing a plain text brief guide explaining how the two main files are organized and interlaced.

## 3 Experimental Design, Materials and Methods

**Fig. 2:**
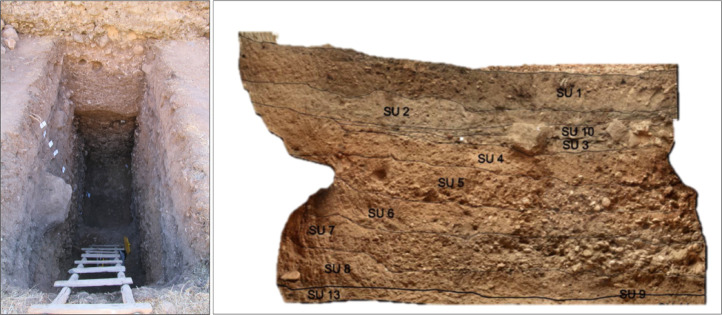
Example of archaeological excavation. The field photograph and the orthogonal section represent Khinis canal site 133. This picture is adapted from Figure 3 in the original publication; for the full stratigraphic characterization, see [1].

Soil samples destined to thin section micromorphology were collected during archaeological fieldwork carried out in the Kurdistan Region of Iraq. Stratigraphic sections cleared during archaeological excavations (Fig. 2) were sampled for micromorphology according to the recorded stratigraphy, addressing notable features that required further investigation. Sampling was carried out by carving the stratigraphic sections to obtain undisturbed and oriented blocks of soil that were later destined for thin section manufacturing. This was carried out by Dr. Massimo Sbrana's “Servizi per la Geologia” laboratory (Piombino, Italy), following the resin consolidation, slicing, mounting and thinning procedure described by Murphy [3]. The finished thin section product is a 30μm thick, 55 × 95mm wide slice of consolidated soil mounted on a glass support and covered with a thin glass protection.

The thin sections were observed employing an optical petrographic microscope (Olympus BX41) mounting a digital camera (Olympus E420) for image acquisition. Observation was carried out at various magnifications (20x, 40x, 100x, 400x) under Plane Polarized Light (PPL) and Cross-Polarized Light (XPL).

Micrographs (Fig. 1A) were taken both as PPL and XPL shots whenever features such as mineral/organic macroscopic components and pedological/sedimentological figures were deemed potentially diagnostic of formation processes for each stratigraphic context that the thin section samples represented.

Captions contained in the “Micrograph captions.pdf” (Fig. 2B) of the produced dataset were created following the guidelines and terminology suggested by Stoops [4], with the aid of the coloured atlases created by Nicosia & Stoops [5], Verrecchia & Trombino [6], and Stoops et al. [7].

## Ethics Statements

No human subjects, animal experiments or data collections from social media platforms were involved in the creation of this dataset. Archaeological fieldwork permits were issued by the General Directorate of Antiquities of the Kurdistan Regional Government, the Directorate of Antiquities of Dohuk, and the State Board of Antiquities and Heritage in Baghdad.

## CRediT authorship contribution statement

**Stefano Costanzo:** Conceptualization, Methodology, Data curation, Visualization, Writing – original draft. **Luca Forti:** Conceptualization, Writing – review & editing. **Daniele Morandi Bonacossi:** Supervision, Funding acquisition. **Andrea Zerboni:** Conceptualization, Methodology, Writing – review & editing, Supervision.

## Declaration of Competing Interest

The authors declare that they have no known competing financial interests or personal relationships that could have appeared to influence the work reported in this paper.

## Data Availability

A thin section micromorphology photomicrographs dataset of the infilling of the Sennacherib Assyrian canal system (Kurdistan Region of Iraq) (Original data) (Zenodo).
